# Comparison of infiltration (INF) and inferior alveolar nerve block (IANB) injection techniques in bilateral therapeutic removal of mandibular premolars

**DOI:** 10.34172/joddd.2021.044

**Published:** 2021-12-05

**Authors:** Balamurugan Rajendran, Sahana Pushpa Thaneraj

**Affiliations:** Department of Dentistry, RYA COSMO Foundation, Chennai, India

**Keywords:** Inferior alveolar nerve block, Infiltration, Mandibular premolar, Therapeutic extraction

## Abstract

**Background.** The present study aimed to evaluate and compare the anesthetic effect of infiltration (INF) and inferior alveolar nerve block (IANB) techniques for bilateral therapeutic extraction of mandibular premolars.

**Methods.** One hundred patients requiring bilateral therapeutic removal of mandibular premolars were included in the study. For the extraction of the mandibular right premolar tooth, INF was used, and after one week, the mandibular left premolar tooth was extracted using the IANB. The effect of anesthesia between the two techniques was compared and evaluated by ANOVA using SPSS.

**Results.** INF was successful in 78% of cases, whereas IANB was successful only in 22% of cases. Furthermore, INF had a significantly better anesthetic effect than IANB (*P* < 0.05). During pain assessment during the anesthetic drug injection and the procedure, two patients in the INF and five patients in the IANB group reported minimal pain during extraction (*P* > 0.05). The onset of the anesthetic effect was faster in the INF group, while the duration of the effect was longer in the IANB group.

**Conclusion.** INF was a more efficacious local anesthetic technique with high success rate than the IANB technique.

## Introduction

Injection techniques for the extraction of posterior mandibular teeth are important for oral and maxillofacial surgeons in controlling and eliminating pain during dental procedures. The inferior alveolar nerve block (IANB) injection technique has been widely used over the past years to extract posterior mandibular teeth, as it is believed that the thick cortical bone of the mandible impedes adequate diffusion of the anesthetic solution. However, the IANB technique is associated with numerous serious complications, such as nerve damage, transient facial paralysis, hematoma, trismus, and increased duration of anesthesia, with possible injuries to the lip and tongue.^1^ To avoid such complications, researchers have constantly worked towards alternate injection techniques with a similar anesthetic effect as that of IANB. Some of such techniques are the periodontal intraligamentary injection (PDL) and local infiltration (INF).^2^ Although PDL injection has a rapid onset of action, the duration of the anesthetic effect is much shorter but inadequate to perform dentoalveolar surgical procedures in the mandible.

In the maxilla and anterior mandible, locally infiltrated anesthesia (INF) has been reported to provide the successful anesthetic effect required for surgical treatments. This is possible due to the presence of trabecular bone at these anatomical sites. However, INF has not commonly been used for surgical procedures in the posterior mandible as it has a dense cortical bony architecture at this site. Some studies^3,4^ have shown that local INF has a successful anesthetic effect in the posterior mandible, with the success rate ranging from 54% to 94%.^3,4^

In the present study, the efficacy of local INF was compared with that of the IANB injection technique for the therapeutic extraction of bilateral mandibular premolar teeth.

## Methods

### Patient selection

This prospective study was carried out on 100 healthy participants >15 years of age, who reported to the Department of Oral and Maxillofacial Surgery for the therapeutic removal of bilateral mandibular premolars. The study protocol was reviewed and approved by the Ethics Committee (EC2018005) and conducted under the Helsinki Declaration. Informed consent was obtained from all the participants.Inclusion criteria were patients >15 years of age, requiring bilateral therapeutic extraction of vital mandibular premolars for orthodontic treatment, and patients with general medical conditions not contraindicated for oral surgical procedures (American Society of Anesthesiologists, ASA-I or ASA-II). Exclusion criteria consisted of patients with known allergies to local anesthetics, pregnant women, patients with active infection or pus at the injection site.

### Treatment protocol

Mandibular right premolar was administered with 2 mL of local INF (1 mL on the buccal side and 1 mL on the lingual side), and the extraction was performed. After one week, the mandibular left premolar underwent IANB with 2 mL of the anesthetic agent (1 mL for the inferior alveolar nerve and 1 mL for the lingual nerve) using a 2-mL cartridge containing 2% lidocaine hydrochloride with 1:80000 epinephrine, and the extraction was performed. No topical anesthetic agent was used before the injection, and the procedure was performed by the same oral surgeon.

### Assessment and follow-up

The following parameters were assessed during and after the removal of mandibular premolars. The onset of anesthetic action was recorded in minutes with a stopwatch from the time of withdrawal of the needle from the injection site to the time when the patient started to experience numbness. The duration of anesthesia was documented in minutes from the time patient started perceiving anesthesia to the moment when the numbness began to fade. The success of anesthesia was checked subjectively (verbal) by asking the patient about the numbness in the anesthetized region. An objective test was also carried out with a probe to the depth of the gingival margin, and the reaction of the patient was noted. Pain during injection and during removal of mandibular premolar was assessed using a 10-cm visual analog scale (VAS), with a score range of 0‒10 with 0 indicating no pain, 5 indicating moderate pain, and 10 signifying the worst possible pain. The duration of anesthesia and patient compliance for both injection techniques were assessed one day after removing the mandibular premolar.

### Data analysis

The significance of differences between INF and IANB was calculated using SPSS 14.0 (Chicago, USA). Descriptive statistics included means, standard deviations, and analyses with ANOVA to assess the significance of differences between INF and IANB. Statistical significance was set at *P* < 0.05.

## Results

Among these 100 patients, 58 were male, and 42 were female, with a mean age of 17 years. Comparisons between the two injection techniques, i.e., INF and IANB, were carried out to assess pain during injection and during extraction of mandibular premolars, the onset of the anesthetic effect and its duration, the efficacy of anesthesia, and its success rate. The data were then analyzed with ANOVA using SPSS (Table 1).

**Table 1:** ANOVA for two injection techniques: infiltration (INF) and inferior alveolar nerve block (IANB) with various parameters: pain during injection and extraction of mandibular premolar, onset and duration of anesthesia, the efficacy of anesthesia, the success rate of anesthesia

**Parameters**	**Infiltration**		**Inferior alveolar nerve block**		***P*** ** value**
	**Mean**	**SD**	**Mean**	**SD**	
Pain during Injection	1.85	0.59	7.21	0.83	0.001
Pain during extraction	1.45	0.10	2.96	5.04	1.110
Onset of anesthesia (min)	2.02	2.15	5.62	4.72	0.042
Duration of anesthesia (h)	60.52	9.96	121.40	8.58	2.247
Efficacy of anesthesia	58%		42%		0.045
Success rate of anesthesia	78%		22%		0.000

### Comparison of INF and IANB

*Pain during injection:*INF resulted in less pain on injection than IANB with a mean difference of 1.85 ± 0.59 for INF and 7.21 ± 0.83 for IANB, indicating a significant difference (P = 0.001).

*Pain during removal of mandibular premolars:*Pain during removal of mandibular premolar was experienced by two patients when the INF technique was used, whereas five patients reported pain during the procedure when IANB was given. Additional injections were administered for two patients in the INF group and five patients in the IANB group (*P* > 0.05).

*Onset of anesthesia (min):*TheINF technique had a faster onset of action than IANB with a mean difference of 2.52 ± 2.15 for INF and 5.62 ± 4.72 for IANB, indicating a significant difference (*P* = 0.042), respectively.

*Duration of anesthesia:*All the patients were evaluated one day after removing mandibular premolars. Patient-reported pain during tooth extraction under INF was associated with a shorter duration of anesthesia than the tooth extracted under IANB, with a mean difference of 60.52 ± 9.96 for INF and 12.40 ± 8.58 for IANB, indicating no significant difference (*P* > 0.05).

*Anesthetic efficacy:*Assessing the efficacy of anesthesia showed that 58% of patients reported anesthesia by INF to be sufficient, while 42% of the patients reported that IANB provided adequate anesthesia, indicating a significant difference (*P* = 0.045).

*Anesthetic success rate:*Extractions under INF had a 78% anesthetic success rate, while extractions under IANB had a success rate of only 22%, indicating a significant difference (*P* = 0.000).

*Patient compliance:*57% of the patients reported nausea and dizziness on the day of extraction under IANB, which might be because of the longer duration of action of the anesthetic drug. However, there was no such complaint by the patients undergoing extraction under INF.

## Discussion

The present study aimed to evaluate the efficacy of anesthesia for mandibular premolars with two different techniques: local INF and IANB. The most common problem experienced by the clinicians or oral surgeons in day-to-day practice is failure to achieve profound anesthesia in patients undergoing dental procedures.^3^ To avoid technical differences among individuals, the procedure must be standardized; therefore, the same oral surgeon carried out the entire experiment. The most preferred injection technique to anesthetize the mandibular posterior region is the IANB. However, it is frequently associated with failure of anesthesia and few other complications, including nerve or vessel damage.^3-5^ Hence, an alternate method is necessary to anesthetize the mandibular posterior region that can safely replace IANB.

Currently, local INF of anesthesia has proven to render a more straightforward and safe mode of drug administration that has an efficacy similar to that of IANB.^5^ The results of the present study demonstrated that the procedure carried out under local INF was significantly less painful than the procedure under IANB, consistent with a study by Bataineh et al.^6^ However, evaluation of the pain experienced by the patients during the procedure showed that two patients had pain when the tooth was extracted under INF. In contrast, the extraction of the other tooth under IANB was painful in five patients, which was then compensated by administering a supplemental injection. In contrast, Yilmaz et al^7^ described that procedures under IANB were less painful than procedures under INF. In contrast, Thiem et al^3^ reported no such differences in the effect of anesthesia, irrespective of the technique used.

The estimation of the onset of action and duration of anesthesia in the present study showed a significant difference between the two techniques. The onset of anesthesia in the INF technique was 2 minutes, with 5 minutes for IANB, similar to the study by Thiem et al.^3^ The effect of anesthesia lasted longer on the left side, which was as long as 120 minutes on average, along with the presence of nausea and dizziness when IANB was used. However, on the right side, it lasted for an average of 60 minutes with INF. The above results are consistent with El-Kholey,^8^ Sierra Rebolledo et al,^9^ Santos et al,^10^ Colombini et al,^11^ and Gregorio et al.^12^

Evaluation of the efficacy of anesthesia revealed that 58% of the patients found the anesthetic effect produced by the INF technique to be sufficient. However, Thiem et al^3^ achieved a different result, demonstrating that IANB offered 80% efficacy, while INF rendered an efficacy of only 44%. In a study by Yilmaz et al,^7^ the success rate of IANB was 70%, while it was only 60% for the procedures carried out under INF. El-Kohley^8^ reported that INF produced profound anesthesia in 93% of cases, and Corbett et al^13^ achieved success rates of 70.4% when INF was used, which had a direct correlation with the current study where a 78% success was achieved with INF and only a 22% success rate was recorded for IANB. Claffey et al^14^ reported a success rate of only 24% for IANB and recorded a failure rate ranging between 44% and 81% when IANB was used.^15-17^

The INF method for anesthesia has numerous benefits over the IANB. The injection technique for INF is simple and more comfortable for patients; it can be used to achieve hemostasis when required, can obviate collateral innervations, and prevents damage to the nerve trunks. It can be prioritized in patients with clotting disorders and to avoid unwanted internal bleeding. The only limitation of INF is that the duration of anesthesia is shorter compared to IANB. Patients were satisfied and comfortable during the procedure with adequate anesthesia rendered through INF, requiring few or no supplemental injections.

## Conclusion

The results of this study clearly showed that INF should be preferred over IANB as it proved a suitable and efficacious technique to achieve profound local anesthesia for the therapeutic removal of mandibular premolars and because it had a faster onset of action and high success rates.

## Author’s Contributions

BR designed and conducted the study and drafted the manuscript. SPT collected and analyzed the data. Both authors read and approved the final manuscript.

## Acknowledgments

None.

## Funding

No funding was received for this study.

## Competing Interests

The authors declare that they have no conflict of interest.

## Ethical Approval

All the procedures performed in this study, involving human participants, followed the ethical standards of the institutional and/or national research committee and complied with the 1964 Helsinki declaration and its later amendments or comparable ethical standards.
